# Tumor Necrosis Factor Inhibitors

**DOI:** 10.32607/actanaturae.27697

**Published:** 2026

**Authors:** T. V. Tregubchak, S. N. Shchelkunov

**Affiliations:** State Research Center of Virology and Biotechnology “Vector”, Rospotrebnadzor, Koltsovo, Novosibirsk region, 630559 Russia

**Keywords:** TNF, orthopoxviruses, rheumatoid arthritis, CrmB, smallpox

## Abstract

Immune-mediated inflammatory diseases affect a substantial proportion of the global population, and tumor necrosis factor (TNF) plays a central role in their pathogenesis. The most common diseases in clude rheumatoid arthritis (RA), Crohn’s disease, psoriasis, multiple sclerosis, and septic shock. All of these conditions are characterized by excessive production of TNF, which activates downstream signaling pathways contributing to disease development and progression. To improve the quality of life in patients with TNF overproduction, anti-TNF agents such as TNF receptors and monoclonal antibodies are used. However, the availability of these therapies is limited. Therefore, the development of novel, more affordable TNF inhibi tors with comparable efficacy and improved safety remains a pressing issue. This review summarizes recent advances in the development of promising TNF inhibitors, including those derived from orthopoxvirus im munomodulatory proteins.

## INTRODUCTION

Tumor necrosis factor (TNF) exerts pleiotropic effects on cells and plays a central role in the regulation of host defense responses. It recruits leukocytes to in f lammation sites by upregulating adhesion molecule expression, stimulates macrophages, increases vas cular permeability, and activates antigen-presenting cells [1, 2]. However, TNF overproduction contributes to the development of pathological conditions associ ated with chronic inflammation and/or autoimmune reactions, such as rheumatoid arthritis (RA), Crohn’s disease, septic shock, and cachexia [3].

Among TNF-induced diseases, RA has a major so cial impact, affecting as it does approximately 0.5–2% of the adult population, particularly individuals of working age (35–55 years) [4]. The condition is char acterized by both synovial and systemic inflammation; if not adequately treated, it leads to disability, degrad ed quality of life, work impairment, and a substantial economic burden [5]. Until the beginning of the 21^st^ century, a third of RA patients had had to stop work ing within two years of the disease onset due to se vere degradation [6]. Moreover, the life expectancy of patients with severe RA drops by an average of 10 years [7]. In this context, the development of effective therapeutic strategies becomes a priority.

RA management has changed substantially over the past 20–25 years: it has shifted from symptom atic treatment to attempts to arrest disease progres sion. This progress has been largely driven by the introduction of disease-modifying drugs, which have significantly improved outcomes for patients. TNF inhibitors represent a major class of such agents; they account for 75% of the total drug market. The most often used TNF inhibitors are the following recombi nant proteins: Remicade (infliximab; Centocor Ortho Biotech/Schering-Plough), Enbrel (etanercept; Amgen/ Wyeth), and Humira (adalimumab; Abbott). These an tirheumatic agents have a number of drawbacks, in cluding adverse effects that limit drug tolerability, the requirement of long-term treatment, and an increased risk of tumor development. In addition, patients often lose responsiveness to the anti-TNF therapy due to the development of an immune response, which usu ally requires drug replacement [3].

Therefore, there is a need for novel anti-RA agents that maintain efficacy while improving safety and cost-effectiveness. Viral TNF-binding proteins represent a promising research area. Poxviruses, the largest DNA viruses infecting mammals, encode nu merous proteins that target key components of the host immune system. Orthopoxviruses, which in clude the variola (smallpox), monkeypox, and cow pox viruses, are of particular interest due to their ability to produce immunomodulatory proteins that bind various ligands, such as TNF, chemokines, in terferons, and complement system components. As a result of virus co-evolution with humans, viral proteins can interact with the host target proteins with high affinity and specificity. This makes viral proteins attractive as candidates for the develop ment of next-generation agents. A detailed study of a broad range of poxvirus protein homologues is required to fully illuminate their therapeutic poten tial. Immunomodulatory proteins of the variola virus, which are evolutionarily the most adapted to the hu man immune system, seem to be the most promising candidates in that regard [8].

## TUMOR NECROSIS FACTOR AND ITS ROLE IN DISEASE

TNF is a pro-inflammatory pleiotropic cytokine in volved in the complex regulation of inflammatory and immune processes. TNF was first described in the middle 1970s [9].

TNF-producing cells are activated in response to external stimuli (e.g., inflammation), resulting in the synthesis of a 26-kDa polypeptide. This polypeptide is expressed on the cell surface as the transmembrane form of TNF (tmTNF). The soluble form of TNF (sTNF) is generated by proteolytic cleavage between Ala76 and Val77 in TNF with involvement of the me talloproteinase TNF-α–converting enzyme (TACE). This process yields a 17-kDa sTNF and a residual cy tosolic domain [10].

TNF exerts its biological function in both trans membrane and soluble forms. Transmembrane TNF initiates a cascade of biochemical reactions through direct contact between a TNF-producing cell and a receptor-exposing cell, whereas sTNF acts at sites distant from TNF-expressing cells [11].

Trimerization of both forms is required for the de ployment of TNF biological activity and induction of intracellular signaling cascades. Following trimeriza tion, TNF binds to a corresponding receptor, either TNFR1 (p55/CD120a; 55 kDa) or TNFR2 (p75/CD120b; 75kDa) [12, 13, 14, 15].

**Fig. 1:**
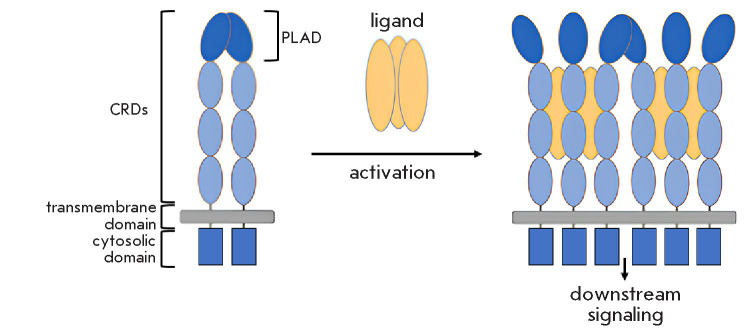
Schematic representation of TNF receptor–ligand interactions. In the absence of a ligand, TNF receptors exist as dimers; upon ligand binding, they form an oligomeric network consisting of trimeric receptor–ligand complexes. The complex formation initiates intracellular signaling [15]. CRDs – Cys-rich domains

The p55 and p75 belong to the TNF receptor (TNFR) superfamily, which comprises at least 29 structurally related type I transmembrane proteins with an extracellular N-terminal and an intracellu lar C-terminal domains; each protein has its distinct structural features [16]. Among these characteristics, cysteine-rich domains (CRDs) are critical for TNFR activity. Each CRD presents a pseudo-repeat of ap proximately 40 amino acid residues containing six Cys residues that form three disulfide bonds with in a single polypeptide chain. The number of CRDs among TNFR family members ranges from one to six. The pseudo-repeats are involved in ligand bind ing [17]. However, not all CRDs participate directly in ligand binding, since TNF interacts primarily with the second and third CRDs from the N-terminus of p55 [18], whereas the first CRD is involved in recep tor oligomerization. This domain is termed the pre ligand assembly domain (PLAD), since it mediates receptor oligomerization at the cell surface prior to li gand binding (Fig. 1). PLAD binding is highly specific, which allows for selective receptor recognition within the TNFR superfamily [19, 20, 21].

Signal transduction following ligand binding de pends on the type of cytosolic domain present in the receptor. The cytosolic domains of TNFRs are rela tively short and function as docking sites for signaling molecules [18]. Receptors can be divided into three groups based on the cytosolic domain structure: death domain-containing receptors (p55), receptors lacking a death domain (p75), and soluble receptors (both p55 and p75 can exist in membrane-bound and soluble forms) [21].

**Fig. 2:**
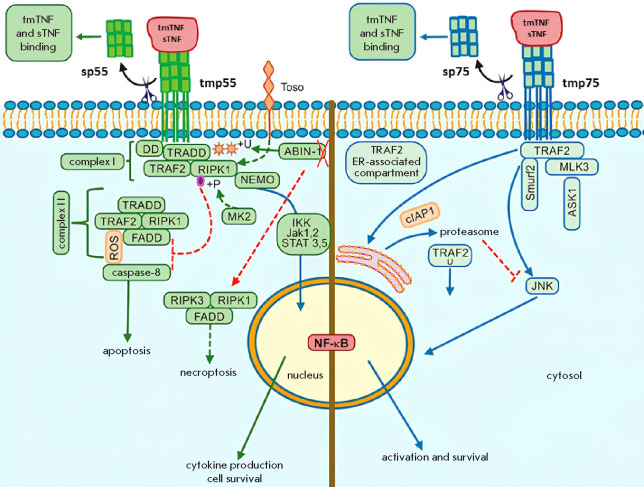
Intracellular signaling pathways activated by TNF binding to p55 and p75. TNF binding to p55 induces cell death and inflammatory signaling through activation of the canonical NF-κB pathway. TNF binding to p75 activates both canon ical and non-canonical NF-κB pathways. Solid green and blue lines represent activating signals; and dashed lines indicate inhibitory interactions. sTNF – soluble TNF; tmTNF – transmembrane TNF; sp55 – soluble p55; tmp55 – transmembrane p55; sp75 – soluble p75; tmp75 – transmembrane p75 [22]

Current evidence indicates the existence of sev eral major signaling pathways. One of the classifi cations is based on the type of receptor the ligand interacts with: there are p55- and p57-mediated path ways (Fig. 2) [22]. It has been previously considered that cytotoxic effects are primarily mediated by p55, while proliferative responses are induced by p75 [23]. However, both p55 and p57 are now known to mediate a broad range of biological functions.

TNF binding to p55 initiates intracellular signal ing through recruitment of adaptor molecules to the death domain of the cytosolic region of the recep tor. As a result, the membrane-associated complex I is formed, which includes TRADD (p55-associated death domain protein), RIPK1 (receptor-interacting protein kinase 1), and TRAF2 (TNFR-associated fac tor 2) [24]. TRAF2 can be recruited to complex I in association with cIAP1 and cIAP2 (E3 ubiquitin ligas es) [25]. These ligases further modify various compo nents of the p55 signaling cascade, including RIPK1, via Lys63-linked ubiquitin chains, thus creating bind ing sites for the linear ubiquitin chain assembly com plex (LUBAC) [26]. LUBAC further modifies RIPK1 with linear ubiquitin chains, facilitating recruitment of the transforming growth factor β-activated kinase 1 (TAK1) via TAB2 (TAK1-associated binding pro tein 2) and IKK (inhibitor of nuclear factor kappa B (κB) kinase complex) [25]. This cascade activates the canonical NF-κB pathway, thus initiating the tran scription of various NF-κB-regulated targets (cyto kine and anti-apoptotic genes). Thus, membrane-asso ciated complex I promotes cell survival mechanisms. If dissociated from the membrane, complex I can form cytosolic complex II through association with FADD (Fas-associated death domain protein) and cas pase-8. Depending on caspase-8 activity, the resulting complex can induce either apoptosis or necroptosis [24, 27].

Similar molecular cascades can be triggered in cells upon TNF binding to p75. However, a central role in p57-mediated signaling is attributed to the c-Jun N-terminal kinase, which, once activated via TRAF2 and ASK1 (apoptosis signal-regulating kinase 1), me diates NF-κB activation [28]. In addition, the p57 sig naling pathway is closely linked to endoplasmic re ticulum (ER) stress. The ER is sensitive to disruptions in cellular homeostasis that lead to the accumulation of misfolded proteins, thereby inducing ER stress and contributing to apoptotic cell death. Under ER stress conditions, TRAF2 associates with ER stress sensors and interacts with pro-caspase-12, promoting activa tion of the latter. It has also been hypothesized that ER stress induces TNF production. This suppresses the c-Jun transcription factor activation and affects cellular susceptibility to apoptosis. These findings con f irm the role of the ER as an additional regulatory node in p57-mediated apoptosis [22].

Thus, the data above indicate that p55- and p75 mediated signaling pathways are closely intercon nected. TNF and its receptors form a complex signal ing network, as indicated by additive, synergistic, and even antagonistic interactions between the two recep tor types [29].

As a key homeostasis regulator, TNF plays an im portant role in conditions of disrupted homeostasis by exerting its pathogenic function. Under normal conditions, TNF contributes to bone tissue regenera tion by maintaining the balance between osteoblasts and osteoclasts; under pathological conditions, it pro motes osteoclastogenesis [2]. TNF suppresses tumor formation under normal conditions and contributes to immune evasion by tumor cells in pathology [1]. Despite numerous examined examples, the mecha nisms underlying the transition to pathological TNF signaling remain unclear. The majority of studies of TNF describe the existing pathologies and only make attempts to establish the underlying causes.

Considering the ubiquitous nature of TNF effects, the spectrum of associated diseases is broad. Major TNF-associated pathologies include rheumatoid ar thritis (RA), Crohn’s disease, septic shock, psoriasis, sarcoidosis, cachexia, Sjogren’s syndrome, polymyo sitis, vasculitis, Behçet’s disease, atherosclerosis, and multiple sclerosis. These conditions are associated with TNF overproduction, although the etiology of the majority of them remains not fully understood [3]. In this review, we focus on three well-characterized TNF-driven diseases.

Among such pathologies, rheumatoid arthritis (RA) is the one that has most extensively been studied. RA is a chronic autoimmune disease that is primar ily accompanied by synovitis, cartilage degradation, and erosive bone damage [30, 31]. RA has a signifi cant social impact, because it is detected in approx imately 0.5–2% of the adult population of working age (35–55 years) [4, 32]. The condition is character ized by both synovial and systemic inflammation and leads to disability, deteriorated quality of life, work impairment, and substantial economic burden, if not adequately treated [5]. Until the beginning of the 21^st^ century, one third of RA patients had to stop working within two years of disease onset [6]. Moreover, the life expectancy of patients with severe RA is lower by an average of 10 years [7]. The RA pathogenesis involves a set of complex interactions of genetic, epi genetic, environmental, metabolic, immune, and mi crobial factors [33, 34]. TNF is a key pro-inflammatory cytokine involved in RA pathogenesis.

In early stages of disease development, initiation of the inflammatory process is accompanied by accumu lation of T helper 1 (Th1) cells, macrophages, B cells, plasma cells, and dendritic cells (DCs). Macrophages and T cells represent major TNF sources at inflam mation sites [35]. Together with interleukin-1 (IL-1), TNF promotes fibroblast proliferation and induces the synthesis of the pro-inflammatory mediators IL-6 and IL-8, the granulocyte–macrophage colony-stim ulating factor (GM–CSF), as well as adhesion mole cules and proteases (e.g., collagenases). Proteolytic enzymes degrade collagen and proteoglycans, resulting in cartilage and bone destruction, with further joint erosion. GM–CSF interacts with TNF through a posi tive feedback pathway and enhances the expression of human leukocyte antigen–DR isotype on antigen presenting cells, thereby promoting T cell activation. TNF stimulates the proliferation of synovial and cir culating T and B cells and upregulates adhesion mol ecule expression on endothelial cells, contributing to T cell adhesion and tissue migration. TNF is also as sociated with osteoporosis in RA; it promotes osteo clast differentiation and stimulates estrogen depletion. Altogether, these processes lead to bone tissue loss. Thus, TNF exerts pleiotropic effects on all the key cell types involved in RA pathogenesis: fibroblasts, endothelial cells, T cells, etc., which proves its central role in disease development. The crucial role of TNF is also confirmed by the clinically effective therapeu tic inhibition of both tmTNF and sTNF in RA patients [36, 37].

Psoriatic arthritis (PsA) shares similar pathogenet ic mechanisms. PsA typically develops in individuals with cutaneous psoriasis and commonly manifests as swelling of the fingers and toes [38]. The condition is characterized by activation of immune cells in in f lamed tissues. Activated DCs and macrophages over express TNF and interleukin-23 (IL-23), which pro mote T cell priming. In PsA, activated T cells further differentiate into effector immune cells, particularly Th17 cells, which overproduce interleukin-17 (IL-17). Together with macrophages, IL-17 plays a key role in joint inflammation and destruction. Macrophages trigger molecular processes similar to those observed in RA [35, 39, 40]. IL-17 and TNF also activate kera tinocytes, promoting epidermal hyperplasia and re cruitment of inflammatory cells, including DCs. TNF induces keratinocyte proliferation and inhibits cell apoptosis via the NF-κB signaling pathway, contribut ing to microabscess formation due to the recruitment of inflammatory cells [35]. Thus, PsA is characterized by changes in the ratio of pro- and anti-inflammatory immune cells and cytokines, thus leading to inflam mation and tissue damage. Numerous studies con f irmed that TNF, IL-23, and IL-17 play a key role in PsA pathogenesis, since their therapeutic inhibitors exert significant therapeutic effects [38, 41].

Sepsis and septic shock represent another socially significant TNF-driven pathology and are responsible for more than 1,000,000 deaths worldwide annually. The mortality rate in septic shock is approximately one out of four cases [42]. Sepsis is a severe clini cal syndrome associated with a dysregulated host re sponse to infection. Its severity is driven by the acti vation of intracellular molecular cascades that lead to amplified cytokine production: the so-called “cytokine storm”, characterized by overexpression of TNF, IL-1, IL-6, IL-8, IL-12, and others. In this condition, patho genic microorganisms are recognized by the innate immune system, which includes leykocytes, the com plement system, cytokines, chemokines, and the an timicrobial peptides secreted by innate immune cells [43].

**Fig. 3:**
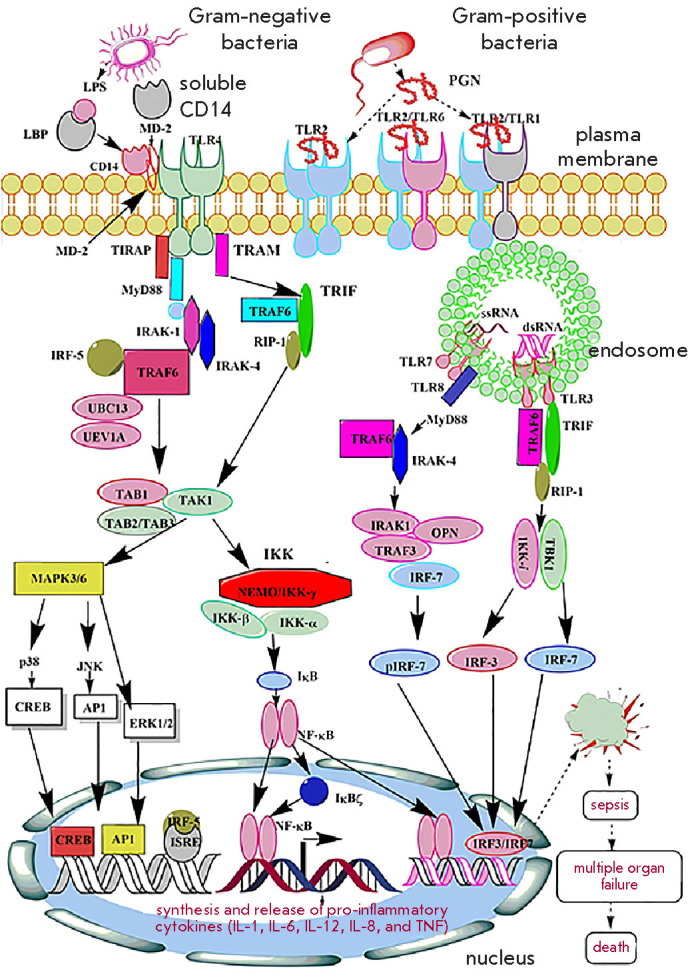
Schematic representation of the activation of vari ous TLR-mediated signaling pathways leading to cytokine storm and sepsis. Activation of these signaling cascades induces the synthesis of pro-inflammatory cytokines (IL-1, IL-6, IL-12, IL-8, and TNF), anti-inflammatory cytokines (IL-10), and type 1 interferons. Elevated levels of these in flammatory mediators lead to cytokine storm in sepsis [44]

The innate immune response is activated through the recognition of pathogen-associated molecular patterns (PAMP), including highly conserved anti gens, such as lipopolysaccharides and peptidoglycans, by pattern recognition receptors (PRR). PRR recognize molecules released from injured tissues (damage-associated molecular patters, DAMP). Four types of PRR are identified in vertebrates: Toll-like receptors (TLR), Nod-like receptors (NLR), RIG-like receptors (RLR), and C-type lectin receptors (CLR). Among these, TLR signaling pathways are the ones that have been most extensively studied. Activation of these signaling cascades induces the NF-κB mediated expression of the pro-inflammatory cyto kines TNF and IL-1β, interferon regulatory factors 3 (IRF3) and 7 (IRF7), as well as adaptor protein 1 (AP-1) (Fig. 3) [43, 44]. Excessive cytokine produc tion results in generalized endothelial activation, in creased expression of adhesion molecules, activa tion of coagulation pathways, and further production of pro-inflammatory cytokines, together ultimately leading to septic shock [45]. Current treatment strat egies in sepsis involve immunomodulatory agents and antibiotics. The potential use of cytokine inhibi tors such as TNF-targeting antibodies are being ac tively explored for disease treatment [46].

These three diseases present overlapping molecu lar pathways and involve TNF overproduction. Hence, similar therapeutic approaches can be used for their treatment. Novel TNF inhibitors can be utilized to treat not only RA, PsA, and septic shock, but also oth er TNF-driven diseases (Table 1).

**Table 1:** Pathogenic role of TNF in a series of autoimmune diseases

Disease	TNF role	Reference
Rheumatoid arthritis	Promotes inflammation by stimulating the recruitment of neutrophils, monocytes, and lympho cytes into the synovial tissue; enhances the secretion of pro-inflammatory cytokines; increases protease production, contributing to joint destruction; stimulates osteoclasts, leading to increased bone resorption and erosive joint damage; contributes to systemic manifestations such as fatigue, anemia, etc.; amplifies disease progression through interaction with other cytokines	[35, 37, 47]
Crohn’s disease	Promotes inflammatory cell infiltration and activation of macrophages and lymphocytes, leading to mucosal injury, erosions, and ulceration; stimulates the production of other pro-inflammatory cytokines, chemokines, and enzymes, thereby enhancing intestinal inflammation; contributes to the formation of granulomatous inflammatory lesions	[48, 49]
Psoriasis	Induces keratinocyte hyperproliferation; promotes the recruitment of immune cells to the skin, thereby enhancing inflammatory cascades and maintaining chronic inflammation; stimulates the synthesis of other cytokines, promoting hyperkeratosis, vascularization, and inflammation	[50, 51, 52]
Psoriatic arthritis	Activates synovial cells, macrophages, T cells, and other immune cells, thereby enhancing syno vial inflammation; promotes activation of enzymes degrading cartilage and bone tissue, leading to joint destruction; increases vascular permeability and stimulates angiogenesis, contributing to inflammatory response and cell infiltration; promotes synthesis and secretion of other pro-in flammatory cytokines, sustaining a chronic inflammatory cycle; stimulates osteoclasts, resulting in bone destruction and joint erosion	[53, 54]
Sepsis	Promotes the development and progression of systemic inflammatory responses, vascular disorders, hypotension, and organ dysfunction	[43, 44, 45]
Cachexia	Activates the destruction of muscle and other tissues, thus contributing to muscle atrophy; enhances basal metabolic rate and insulin resistance and triggers lipid metabolism disorders, contributing to fat loss and energy imbalance; suppresses appetite centers in the hypothalamus, resulting in anorexia; sustains a chronic inflammatory state	[55, 56]

## DRUGS INHIBITING THE BIOLOGICAL FUNCTION OF TNF

The rapid development of molecular biology tech niques, together with the establishment and investi gation of experimental models of immune-mediated inflammatory diseases in the second half of the 20th century, enabled the establishment of the crucial role of TNF in the pathogenesis of a series of conditions, including RA, Crohn’s disease, ulcerative colitis, and septic shock.

Some of the earliest evidence for the TNF role in inflammatory arthritis was obtained in transgenic mice overexpressing human TNF (hTNF). These mice spontaneously develop chronic inflammatory polyarthritis, which was stopped by administration of hTNF-specific monoclonal antibodies [57]. It is now well established that TNF is a key effector molecule in the inflammatory cascades underlying many auto immune diseases [58]. Advances in genetic engineer ing have enabled the generation of mouse models for studying the pathogenesis of such diseases as colla gen-induced arthritis, autoimmune encephalomyelitis, colitis, bacterial infections, etc. The use of these mod els confirmed the role of TNF in disease development [59].

The results of this work served as the basis for nu merous studies into the development of TNF inhibi tors for the treatment of immune-mediated diseases such as RA and Crohn’s disease.

The primary objective of an anti-TNF therapy is to inhibit TNF-mediated signaling pathways and, there by, suppress the inflammatory response. In this re gard, therapeutic strategies targeting various mol ecules involved in TNF synthesis and processing have been developed. Potential pharmacological targets include intracellular signaling molecules such as ki nases, transcription factors, and molecules involved in mRNA splicing, translation, and protein maturation [60]. However, many of these targets are pleiotropic, which limits the design of selective inhibitors [61]. Therefore, the most effective strategy seems to be to suppress the interaction between TNF and its recep tors, thereby rendering the downstream signal trans duction impossible [60]. To date, the strategy based on the use of TNF inhibitors remains the predominant approach.

In the late 1990s, TNF inhibitors emerged as a revolutionary therapy against immune-mediated inflammatory diseases affecting joints, the gastroin testinal tract, and skin. Numerous clinical trials dem onstrated the efficacy and safety of these drugs in large cohorts of patients. In 1998, the Food and Drug Administration (FDA, USA) approved the first TNF targeted drugs based on TNFR and a monoclonal an ti-TNF antibody for the treatment of RA and Crohn’s disease, respectively [62].

**Fig. 4:**
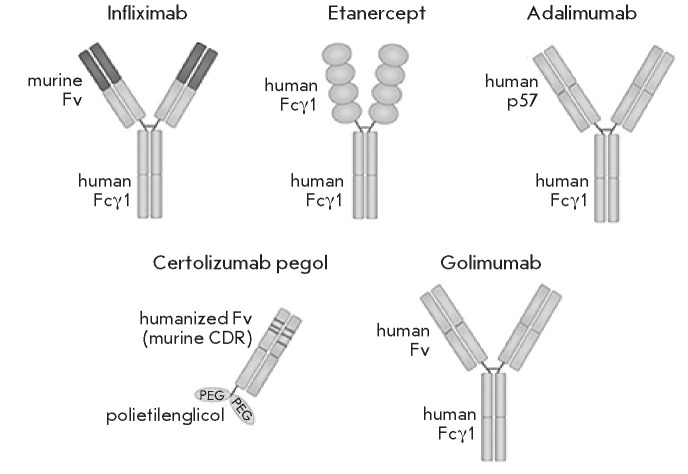
Simplified representation of the molecular structures of TNF antagonists. Infliximab is a chimeric mouse/human IgG1 monoclonal anti-TNF antibody. Adalimumab and golimumab are fully human IgG1 monoclonal anti-TNF antibodies. Etanercept is a fusion protein consisting of two extracellular domains of human p75 and the Fc region of human IgG1. Certolizumab pegol is a PEGylated Fab fragment of humanized IgG1 monoclonal anti-TNF antibody [64]

There are currently five major anti-TNF drugs in clinical use: the monoclonal anti-TNF antibodies in f liximab, adalimumab, golimumab, and certolizumab pegol; and a TNFR-based drug, etanercept (Fig. 4) [63, 64]. Other anti-TNF agents present analogues of the above-listed drugs. The analogues have been de veloped primarily to improve therapeutic accessibility and reduce production costs [65, 66].

Etanercept (Enbrel) was the first anti-TNF drug approved for RA treatment in 1998. It consists of a recombinant protein consisting of two extracellu lar domains of human p75 linked to the Fc fragment of human IgG1 (Fig. 4). The recombinant protein is produced in Chinese hamster ovary (CHO) cells [3]. The presence of the Fc region in the drug prolongs the circulating half-life of the molecule, contributing to its long-term therapeutic effect [67]. Etanercept has a broad range of clinical applications: RA, PsA, and non-radiographic axial spondyloarthritis (Table 2) [65, 68].

Infliximab was approved by the FDA in 1998 for the treatment of Crohn’s disease. The drug repre sents a chimeric monoclonal antibody and consists of a human IgG1 light chain constant region and a vari able region of a murine anti-TNF antibody (Fig. 4). The antibody is obtained using hybridoma technology and produced in recombinant cell systems cultured by continuous perfusion [69]. Although infliximab is highly specific and exhibits minimal adverse effects on nontarget biological pathways, the presence of a murine antibody fragment in its structure may trig ger an immune response [70]. The drug is used for the treatment of Crohn’s disease, RA, PsA, ulcerative colitis, and other pathologies (Table 2) [71].

**Table 2:** Indications to the use of TNF inhibitors and dates of the first clinical approval (by the FDA and the European Medicines Agency (EMA)) [68]

Drug	Disease	Clinical approval year
Etanercept	Rheumatoid arthritis	1998
	Juvenile idiopathic arthritis	1999
	Psoriatic arthritis	2002
	Axial spondyloarthritis	2003
	Plaque psoriasis	2004
	Pediatric plaque psoriasis	2008
	Non-radiographic axial spondyloarthritis	2014
Infliximab	Crohn’s disease	1998
	Rheumatoid arthritis	1999
	Axial spondyloarthritis	2003
	Psoriatic arthritis	2004
	Plaque psoriasis	2005
	Ulcerative colitis	2005
	Pediatric Crohn’s disease	2006
	Pediatric ulcerative colitis	2011
Adalimumab	Rheumatoid arthritis	2002
	Psoriatic arthritis	2005
	Axial spondyloarthritis	2006
	Crohn’s disease	2007
	Plaque psoriasis	2007
	Juvenile idiopathic arthritis	2008
	Ulcerative colitis	2012
	Pediatric Crohn’s disease	2012
	Non-radiographic axial spondyloarthritis	2012
	Pediatric enthesitis-related arthritis	2014
	Pediatric plaque psoriasis	2015
	Hidradenitis suppurativa	2015
	Adolescent hidradenitis suppurativa	2016
	Non-infectious uveitis	2016
	Pediatric non-infectious uveitis	2017
	Pediatric ulcerative colitis	2020
Golimumab	Rheumatoid arthritis	2009
	Psoriatic arthritis	2009
	Axial spondyloarthritis	2009
	Ulcerative colitis	2013
	Non-radiographic axial spondyloarthritis	2015
	Juvenile idiopathic arthritis	2016
Certolizumab pegol	Crohn’s disease	2008
	Rheumatoid arthritis	2009
	Psoriatic arthritis	2013
	Axial spondyloarthritis	2013
	Non-radiographic axial spondyloarthritis	2013
	Plaque psoriasis	2018

Adalimumab is a human IgG1-based monoclonal anti-TNF antibody produced in CHO cells (Fig. 4). It was approved by the FDA in 2002 for the treatment of RA and became one of the top ten most widely used monoclonal antibodies in 2018 and, subsequently, the most profitable drug worldwide [72]. Later, adali mumab was also approved to treat other immune-me diated inflammatory diseases such as Crohn’s disease, plaque psoriasis, and ulcerative colitis (Table 2).

Golimumab is another human IgG1-based monoclo nal anti-TNF antibody. Unlike adalimumab, which is obtained using phage display technology, golimumab was produced using transgenic mice expressing hu man immunoglobulin genes. These mice were immu nized with human TNF; clones producing high-affinity anti-hTNF antibodies were generated using the hy bridoma technology [73, 74]. Golimumab has now been approved for the treatment of RA, PsA, and other in f lammatory conditions (Table 2) [75].

Certolizumab pegol is a monovalent Fab frag ment of a humanized anti-TNF IgG antibody lacking the Fc region [76]. It is the absence of the Fc region that distinguishes the drug from other TNF inhibi tors. This structural feature would normally result in a more rapid drug clearance, since the interaction between the Fc region and the neonatal Fc receptor in the endosome is important in the regulation of an tibody homeostasis by protecting IgG from degrada tion, thereby prolonging its plasma half-life. However, the plasma half-life of certolizumab pegol is already extended through the conjugation of the hinge region of the Fab fragment to two interlinked 20-kDa poly ethylene glycol (PEG) moieties. Such a modification increases the drug half-life and solubility, while re ducing its immunogenicity and sensitivity to proteo lytic degradation [77]. The drug was first approved in 2008 for Crohn’s disease and later for such conditions as RA, PsA, and others (Table 2).

Although all TNF inhibitors neutralize the same molecular target, clinical data (summarized in Table 2) indicate that there are differences in their efficacy across diseases. For example, unlike monoclonal an tibody-based TNF inhibitors (adalimumab and inf liximab), etanercept has not shown any clinically sig nificant efficacy in the treatment of the inflammatory bowel disease or uveitis [78]. Moreover, a significant variability in the individual response to TNF inhibi tors has been observed. While some patients respond rapidly to the drug’s administration, others exhibit ei ther a delayed or no response. In addition, some indi viduals may experience a loss of response to anti-TNF therapy over time [64].

The described differences in therapy effectiveness may be an indication of the existence of individu al polimorphisms of immune-associated genes and highlight the need for further investigation into the molecular basis of disease pathogenesis to develop personalized therapeutic approaches. Disease-specific variations in the clinical efficacy of TNF inhibitors have also prompted discussion regarding potential dif ferences in the mechanisms of action [79].

Studies using surface plasmon resonance have shown that golimumab binds sTNF with affinity com parable to that of etanercept and higher than that of infliximab and adalimumab [74]. Furthermore, the binding avidity of etanercept to sTNF is 10–20-fold higher than that of infliximab or adalimumab [80].

In addition, infliximab, adalimumab, etanercept, go limumab, and certolizumab pegol bind tmTNF ex pressed on the cell surface with comparable affinity [81, 82], although their affinity is lower than that of sTNF [80]. Some studies have reported that etaner cept either binds tmTNF with lower affinity or does not bind it at all, compared to anti-TNF drugs based on monoclonal antibodies [83, 84]. These differenc es may be primarily due to the use of different cell lines with variable levels of tmTNF expression levels. Yet, another literature source indicates that etaner cept forms relatively unstable complexes with sTNF, resulting in faster TNF dissociation, while infliximab binds both tmTNF and sTNF to form more stable high-molecular-weight complexes [85]. Differences in the binding stoichiometry have also been report ed. Infliximab can bind both monomeric and trimeric TNF forms, resulting in stable high-molecular-weight complexes. Each infliximab molecule binds to two TNF molecules, whereas etanercept binds TNF ho motrimers at a 1 : 1 ratio [86]. Taken together with the fact that infliximab forms more stable complexes with tmTNF, this may explain the drug’s clinical ef f icacy in the case of the inflammatory bowel disease and the absence of that for etanercept. Recent stud ies suggest that neutralization of tmTNF, rather than sTNF, is critical for therapeutic efficacy in the inflam matory bowel disease [87, 88].

Thus, having considered only one of the main mechanisms of action of TNF inhibitors, namely TNF neutralization, it seems obvious that differences in ki netic, stoichiometric, and other parameters of ligand receptor interactions contribute to the variability in the therapeutic efficacy of TNF inhibitors in different diseases.

Differences in the pharmacokinetic profiles may also contribute to the variations in the clinical effi cacy of TNF inhibitors. Of all these drugs, etanercept has the shortest half-life (4–5 days), whereas intact IgG1-based drugs exhibit longer half-lives: 8–10 days for infliximab, 10–20 days for adalimumab, 7–20 days for golimumab, and 14 days for certolizumab pegol. In addition, etanercept possesses the lowest steady state concentration: 1.1 µg/mL, compared to infliximab (118 µg/mL) and adalimumab (4.7 µg/mL). Taken to gether, these differences suggest that monoclonal an tibody-based drugs may provide a longer therapeutic effect than etanercept [64].

The pharmacokinetic properties of TNF inhibitors underpin the frequency of drug administration, which ultimately results in increased systemic exposure and production of specific anti-drug antibodies. For ex ample, production of anti-drug antibodies has been reported in approximately 1.2% of patients receiv ing etanercept and 3.8% in cases of golimumab use. Infliximab was shown to be the most immunogenic (25.3% of cases), followed by adalimumab (14.1%), and certolizumab pegol (6.9%) [89]. The immunogenicity of these agents may vary depending on the disease, dos age regimen, and therapy duration [90].

Despite the substantial success of anti-cytokine therapy in the treatment of TNF-driven diseases, this therapeutic approach allows only to affect dis ease symptoms, rather than eliminate the underly ing cause. In addition, the therapy may cause severe adverse effects due to the suppression of the cyto kine protective functions [91]. Reported adverse ef fects include infusion and injection-site reactions, infections (in particular, reactivation of tuberculosis), autoantibody formation and development of drug induced lupus, hepatic dysfunction, as well as hema tologic and solid malignancies [92, 93, 94]. In addition, allergic reactions may emerge due to the production of antibodies to anti-TNF drugs, thus reducing treat ment efficacy [95]. As noted above, immunogenicity varies depending on disease type, dosage, and ther apy duration [90].

A comprehensive meta-analysis has been con ducted for each TNF inhibitor to explore associations between drug administration and adverse effects; indeed, the occurrence of such effects is well docu mented [96]. Novel therapeutic approaches are cur rently under development, including modification of existing agents to reduce the adverse effects. It has been confirmed that the main limitation to the use of TNF inhibitors is related to the pleiotropic roles of the cytokine. For example, TNF production and its involvement in the formation of granulomas, which contain the infection in its inactive form, determine the host defense profile against the tuberculosis in fection. In this regard, TNF inhibitors may lead to the activation of latent tuberculosis [93, 94]. Experimental studies in various disease models have demonstrated that TNF produced by myeloid cells exerts a patho genic effect, while TNF derived from T cells has a protective role. Findings in mouse models using tis sue-specific knockout suggest that, if a potential TNF inhibitor can selectively avoid inhibition of a T cell derived TNF, it may reduce the adverse effects of the therapy [91].

A practical implementation of this hypothesis is the development of bispecific antibodies, referred to as MYSTI. These antibodies are specific to both TNF and the surface markers of myeloid cells, which en ables selective neutralization of TNF produced by my eloid cells. The use of such a drug is expected to re duce the incidence of adverse effects, since earlier experimental studies in mice have demonstrated that it is the TNF derived from myeloid cells that often contributes to disease development, while T cell-de rived TNF exerts a protective function. This targeted inhibition of TNF depending on the origin cell type represents a potential advantage of MYSTI-based therapeutics [97, 98].

We can conclude that the findings of the past 20 years of research on TNF-targeted therapies indi cate that suppression of TNF–receptor interactions remains the most effective therapeutic strategy. In this regard, the majority of current studies aim to modify existing drugs that inhibit the interaction be tween TNF and its receptors, p55 and p75.

## TNF RECEPTORS OF ORTHOPOXVIRUSES

For the past 10 years, there has been accumu lating evidence of the potential of using viral proteins as therapeutic agents. In this context, patho genic orthopoxviruses (genus Orthopoxvirus, family Poxviridae) are of particular interest. These viruses are among the largest DNA viruses. Their replica tion cycle takes place entirely in the cytoplasm [99]. These viruses encode a wide spectrum of immuno modulatory proteins [8]. It is considered that, through co-evolution with the host, these viruses can acquire the encoding sequences of various host genes in their genome and modify them, thus adapting them to viral survival and resilience in the biosphere [99, 100].

Cowpox virus (CPXV) is of low pathogenicity in humans, while having a broad host range among an imals. Monkeypox virus (MPXV) is also character ized by a broad host range; in humans, it causes an infection clinically similar to smallpox and even fa tal in some cases. Variola virus (VARV), which rep resents a rare case of a strictly human-adapted vi rus, is of special interest. VARV is highly pathogenic to humans; it has evolutionarily adapted to efficiently evade the human immune defense [8, 99].

Having sequenced the complete genomes of VARV [101, 102, 103, 104, 105], MPXV [106, 107], and CPXV [108] and performed further genomic analyses, we managed to identify the viral genes encoding proteins of the TNFR superfamily. That was the first experimental evidence that the ST-2 protein of the Shope fibro ma virus and the MT-2 protein of the myxoma virus (poxviruses of the genus Leporipoxvirus), which com prise the TNFR superfamily, bind to TNF [109].

Four genes encoding TNFR proteins were identi f ied in CPXV, while only one such gene was found in VARV and MPXV: CrmB. CrmB has been regarded as a novel, potential TNF inhibitor.

**Fig. 5:**
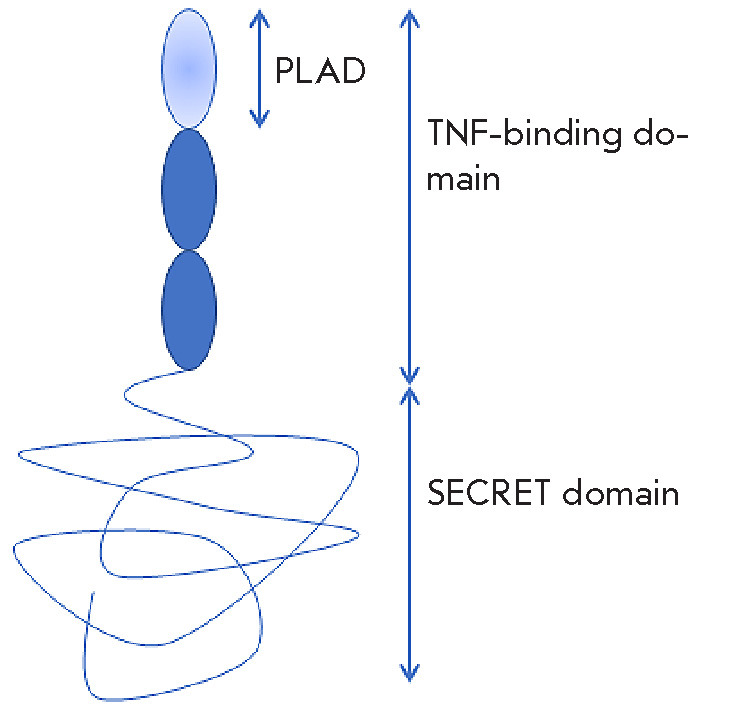
Domain organization of the CrmB protein. Ovals represent the three N-terminal CRDs

CrmB is an early viral protein (i.e. it is expressed in the host cell prior to viral DNA replication) with a molecular mass of approximately 47 kDa. The pro tein is composed of two domains. The N-terminal re gion contains the TNF-binding domain, comprising three CRDs, each containing six Cys residues [108, 110]. A PLAD is located within the first N-terminal CRD (Fig. 5) [111]. The TNF-binding domain shares approximately 37.5% to 40% sequence homology with human p55 and p75 [112]. In addition, CrmB contains a C-terminal chemokine-binding domain, referred to as the SECRET domain (Fig. 5) [113].

**Fig. 6:**
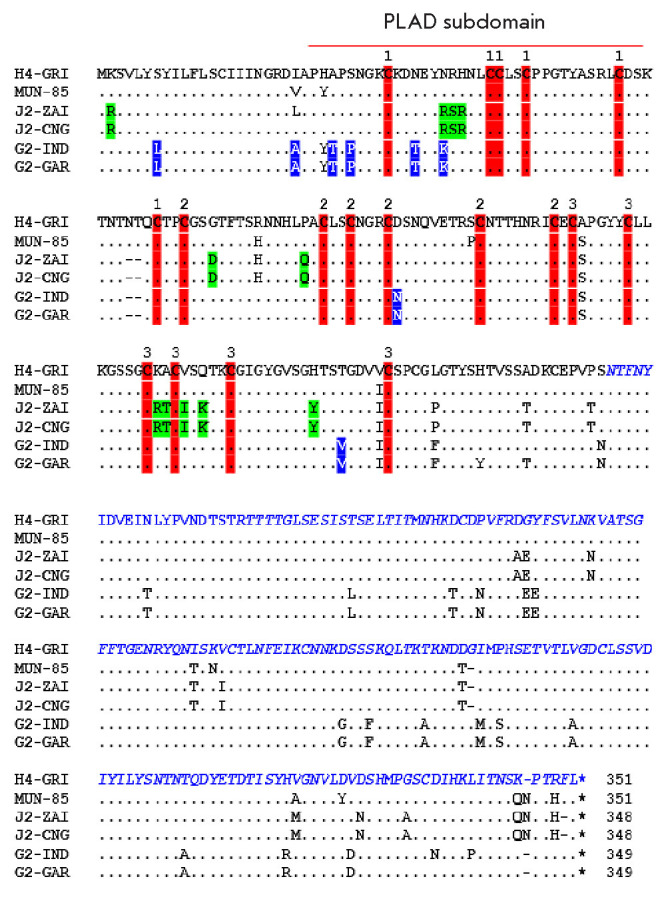
Comparison of the amino acid sequences of or thopoxvirus CrmB proteins belonging to the TNFR super family. Two strains of each virus were compared: CPXV (GRI and MUN-85), MPXV (ZAI and CNG), and VARV (IND and GAR). Amino acid residues identical to CPXV GRI are indicated by dots; deletions are represented by dashes. Species-specific differences between VARV and MPXV/CPXV in the TNFR region are highlighted by blue and green, respectively. The SECRET domain sequence is shown in blue italics. The PLAD subdomain sequence is marked by a red line. Red vertical bars and the cor responding numbers above them denote Cys residues forming the three CRDs

A comparative analysis of the CrmB amino acid se quences of CPXV, MPXV, and VARV has revealed the species-specific differences that may determine the variability of their functional properties (Fig. 6) [114].

We conducted extensive studies to determine the properties of the recombinant full-length CrmB pro teins of VARV, MPXV, and CPXV in order to estab lish the species-specific differences between them. Recombinant proteins were produced using a bacu lovirus expression system; the resulting proteins had a molecular mass of 45–47 kDa. Despite a high de gree of sequence homology (85–96%) between them, species-specific differences were identified in assays evaluating the ability of recombinant orthopoxviruses proteins to bind TNF from different animal species. VARV CrmB was shown to neutralize the cytotox ic activity of both human TNF (hTNF) and murine TF (mTNF) with similar effectiveness in vitro [115], whereas CPXV CrmB effectively neutralized only mTNF, not hTNF [114, 116]. MPXV CrmB did not ex hibit any significant inhibitory effect toward either ligand [114]. Thus, the TNF-neutralizing activity of these proteins varies substantially across animal spe cies, which is likely due to differences in their amino acid sequences.

A gel filtration analysis demonstrated elution of VARV CrmB in the fraction corresponding to a mo lecular mass of > 500 kDa, indicating protein oligo merization, while CPXV CrmB was primarily detected in the fraction corresponding to dimeric forms [112].

Viral immunomodulatory proteins, unlike their cel lular analogues, can form stable oligomers in the ab sence of a ligand, which may enhance efficiency in their binding to target proteins.

It was also shown that CrmB inhibits the binding of hTNF to polyclonal anti-TNF antibodies, with in hibitory effectiveness decreasing in the following or der: VARV CrmB > CPXV CrmB > MPXV CrmB [117]. The hTNF-neutralizing activity of these pro teins in vitro was comparable to that of human p55 and p57, as well as the monoclonal antibody mAb MAK195. The TNF-neutralizing activity of Remicade was slightly higher than that of the two-domain VARV CrmB: at 50% cell viability, the ratio of hTNF to VARV CrmB and Remicade concentrations was 2 : 4–8 and 2 : 0.5–1 ng/mL, respectively [112].

The therapeutic efficacy of recombinant CrmB proteins from VARV, MPXV, and CPXV was further evaluated in a mouse model of lipopolysaccharide (LPS)-induced endotoxic shock. In that experiment, chimeric variants of the VARV and CPXV CrmB pro teins fused to the heavy chain fragment of human IgG1 (hereafter referred to as VARV CrmB/IgG1 and CPXV CrmB/IgG1) were also tested, since such mod ifications increase protein avidity and the circulat ing half-life [118]. As a result, the VARV CrmB and VARV CrmB/IgG1 proteins demonstrated a significant therapeutic impact, unlike CPXV CrmB, CPXV CrmB/ IgG1, and MPXV CrmB. Of these, VARV CrmB/IgG1 exhibited the strongest therapeutic effect [115, 116].

The obtained results indicate that even highly ho mologous CrmB proteins from different orthopoxvirus species can vary significantly in ligand specificity and, in particular, exhibit different biological activities to wards ligands from different species.

Administration of the recombinant VARV CrmB protein to intact mice did not lead to any changes in the animals’ behavior, appearance, or histological structure of internal organs compared to the control mice, suggesting an absence of a toxic effect of the drug under the experimental conditions used [115].

In a number of additional studies, VARV CrmB also demonstrated efficacy as an hTNF antagonist, which led researchers to consider it as a potential platform for the development of novel TNF inhibitors for the treatment of TNF-driven diseases [119].

Since therapeutic agents typically require repeated administration, it is important that the drug possess low immunogenicity. In this regard, we assessed the immunogenic properties of the VARV CrmB upon re peated administration in BALB/c mice. The high im munogenicity of recombinant VARV CrmB was noted, and we speculated that it may be partially associated with the previously described fact of protein multi merization [112, 120].

**Fig. 7:**
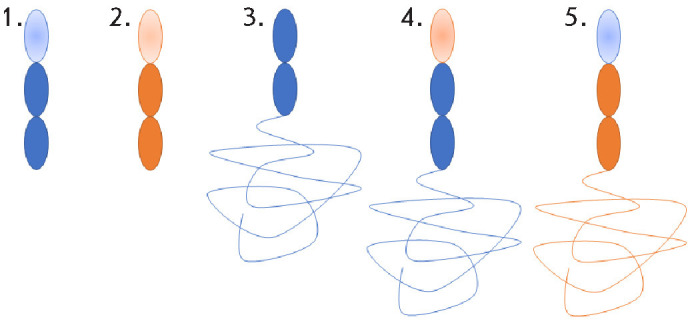
Schematic representation of chimeric and truncated variants of the CrmB protein produced using the baculovi rus expression system. Regions corresponding to VARV CrmB and CPXV CrmB are shown in blue and orange, respectively. 1 – VARV CrmB lacking the SECRET domain. 2 – CPXV CrmB lacking the SECRET domain. 3 – VARV CrmB lacking the PLAD subdomain. 4 – VARV CrmB containing the PLAD subdomain of CPXV CrmB. 5 – CPXV CrmB containing the PLAD subdomain of VARV CrmB

To reduce the immunogenicity and establish the functional significance of individual domains, we generated recombinant deletion and chimeric vari ants of VARV CrmB and CPXV CrmB (Fig. 7). The genes encoding recombinant proteins were expressed using a baculovirus system in Sf-21 insect cells. It was discovered that deletion of PLAD (located in the f irst CRD) from VARV CrmB removed the protein’s ability to neutralize the cytotoxic effect of hTNF in L-929 mouse fibroblasts. Replacement of PLAD in VARV CrmB with that of CPXV CrmB resulted in re duced inhibitory activity towards hTNF (compared to VARV CrmB), while the reverse substitution of PLAD in CPXV CrmB yielded the opposite effect [121].

We also demonstrated that deletion of the SECRET domain did not impair the ability of the truncated CrmB protein (hereafter referred to as the TNF binding domain (TNF-BD)) from VARV to inhibit the cytotoxic activity of hTNF [121].

To evaluate VARV TNF-BD as a potential agent for TNF-targeted therapy we needed further study into its TNF-binding properties in both in vitro and in vivo experiments.

We compared the ability of the VARV TNF-BD and two-domain VARV CrmB proteins to inhibit the TNF interaction with specific cellular receptors. For this, we evaluated their capacity to neutralize the cytotoxic effect of hTNF and mTNF in L-929 cells. The experi ments demonstrated that both proteins inhibit the cy totoxic effect of hTNF and mTNF with comparable efficacy [121].

The biological activity of VARV TNF-BD in vivo was evaluated in a mouse model of LPS-induced endotoxic shock. In mice receiving LPS, mortality reached 90% within 72 h after injection. In contrast, administration of recombinant proteins (both VARV TNF-BD and VARV CrmB) resulted in a survival rate of 62.5% [121].

To minimize the potential cost of VARV TNF-BD production, a E. coli expression system was devel oped. The biological activity of the recombinant VARV TNF-BD protein was assessed based on its ability to neutralize the cytotoxic effects of hTNF and mTNF in L-929 mouse fibroblast cells. The protein produced in the prokaryotic system was shown to retain biological activity and effectively neutralize TNF-induced ef fects in vitro [121].

Furthermore, a comparative analysis of the effects of VARV CrmB and VARV TNF-BD on the TNF induced oxidative and metabolic activity of blood leu kocytes in intact mice showed that removal of the chemokine-binding domain in VARV CrmB does not impair the ability of the truncated, non-glycosylated VARV TNF-BD to neutralize the biological actions of TNF [122].

The obtained results support the potential of the non-glycosylated TNF-BD protein derived from VARV, produced in a bacterial expression system, as a candidate for anti-TNF therapy. In this regard, we further evaluated the immunogenic properties of the truncated TNF-binding protein. Deletion of the che mokine-binding domain in VARV CrmB resulted in a reduced humoral immune response compared to the full-length protein, which may favor the therapeutic application of the truncated protein TNF-BD [120].

Since the truncated VARV TNF-BD protein ex hibits reduced immunogenicity, it can be considered a promising candidate for the development of novel anti-TNF therapeutics. Therefore, the affinity of its interaction with TNF was further investigated.

First, protein–protein interactions between truncat ed CrmB and TNF were analyzed using bioinformat ic modeling [123]. VARV TNF-BD and CPXV TNF BD proteins were chosen as receptors, and hTNF and mTNF were used as ligands. We selected truncat ed CrmB proteins for the experiments, because their full-length analogues, despite high, 87% sequence ho mology, exhibit species-specific differences in ligand binding [114].

**Fig. 8:**
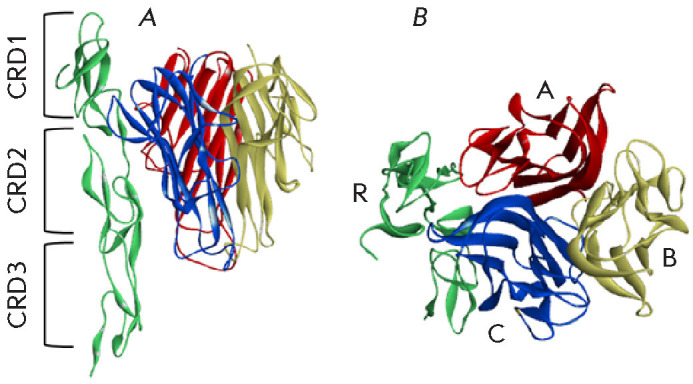
Predicted three-dimensional structure of the com plex of hTNF homotrimer with a CPXV TNF-BD molecule, shown in side (A) and top (B) views. CRDs are indicated by square brackets. Colors and labels correspond to different complex subunits (R – CPXV TNF-BD; A, B, and C – individual units of hTNF homotrimer). The structure is presented as a ribbon diagram [123]

The three-dimensional structures of VARV TNF-BD, CPXV TNF-BD, and TNF were predict ed using the Swiss-model (Fig. 8). The structure of the TNF-binding domain of the human p75 receptor (pdbid: 3ALQ) in a complex with mutant hTNF was used as a template for modeling the three-dimension al protein structures [123].

Next, the efficiency of protein–protein interac tions was evaluated using surface plasmon resonance (SPR) analysis. Dissociation constant (KD ) values were determined for both the full-length proteins VARV CrmB and CPXV CrmB produced in Sf-21 cells and their truncated variants VARV TNF-BD and CPXV TNF-BD generated in E. coli.

**Table 3:** SPR analysis of the effectiveness of interaction between hTNF, mTNF, and viral receptors

TNF antagonist	K_D_, M hTNF	K_D_, M mTNF
VARV CrmB (produced in Sf-21 cells)	2.48 × 10^-9^	3.62 × 10^-10^
VARV TNF-BD (produced in E. coli)	5.26 × 10^-10^	8.63 × 10^-10^
CPXV CrmB (produced in Sf-21 cells)	4.10 × 10^-9^	8.52 × 10^-10^
CPXV TNF-BD (produced in E. coli)	6.46 × 10^-9^	1.16 × 10^-9^

The KD values obtained for the complexes CPXV TNF-BD/hTNF and VARV TNF-BD/hTNF aligned with the results of molecular dynamics simula tions using the MM-GBSA implicit solvation mod el: the free energy of complex formation was high er for VARV TNF-BD/hTNF compared to CPXV TNF-BD/hTNF (Table 3) [123].

In terms of immunogenicity and hTNF-binding ef f iciency, VARV TNF-BD demonstrates potential ad vantages relative to currently available anti-TNF drugs and may become a prospective candidate that can compete with foreign alternatives based on an tibodies and TNFR (Table 4). The binding affinity of VARV TNF-BD for hTNF is either comparable or even higher than that of available alternatives. The lower molecular weight of TNF-BD contributes to its reduced immunogenicity and may allow for usage of lower therapeutic doses. VARV TNF-BD production in E. coli also allows for significantly reduced manu facturing costs compared to protein expression in eu karyotic cells.

**Comparison of anti-TNF agents:** Comparison of anti-TNF agents

Agent	hTNF binding affinity, K_D_, M	Molecular mass, kDa	Expression system used for protein production
VARV TNF-BD	2.48 × 10-9	17	E. coli
Commercial drugs			
Etanercept	4.01 × 10-8	149	mammalian cells
Adalimumab	8.6 × 10-9	149	mammalian cells
Infliximab	4.2 × 10-9	149	mammalian cells

To further curb the immunogenicity, we used an alternative strategy for protein delivery. For this, we constructed a recombinant plasmid (VARV pcDNA/TNF-BD) encoding VARV TNF-BD to enable its expression in mammalian cells. The therapeutic potential of this construct was evaluated in a rat mod el of collagen-induced arthritis (CIA). Injection of the recombinant plasmid in CIA rats resulted in impeded histopathological joint damage compared to the con trol animals receiving the pcDNA vector [124]. These f indings suggest the potential of using gene therapy approaches to treat RA based on local injection of a recombinant plasmid to ensure VARV TNF-BD ex pression.

Repeated administration of a therapeutic dose of VARV pcDNA/TNF-BD induced a specific immune response against the expressed protein in mice; how ever, the response was significantly more muted than that observed upon repeated injection of the recom binant protein [125]. This observation further sup ports the potential advantages of this anti-TNF ap proach.

The collected experimental data indicate that VARV TNF-BD represents a promising platform for the de velopment of novel TNF inhibitors.

Further study of viral TNFR homologues may sig nificantly contribute to the improvement of existing anti-TNF therapies, primarily through expanding our knowledge of the mechanisms of ligand-receptor in teractions.

One such study was performed in 2019. In it, modi f ication of the primary structure of etanercept, in par ticular the introduction of a Glu–Phe–Glu amino acid motif into one of the structural loops, resulted in a 3- and 60-fold reduction in its biological activity against lymphotoxin (LT) and TNF, respectively. Modulating the ability of this potential TNF inhibitor to bind to LT should reduce the incidence of infectious diseases during therapy, since LT is involved in inducing im mune responses in conditions of suppressed TNF sig naling. In addition, the role of LT in inflammatory dis eases remains uncertain; for instance, studies of RA patients have revealed no therapeutic effect from LT targeted antibodies [126, 127].

At the same time, the reduced anti-TNF activity in the modified etanercept variant may adversely af fect its therapeutic efficacy. Such limitation can be mitigated in clinical settings through increasing the dose. Thus, the modified protein may serve as a basis for the development of safer etanercept-like thera peutics.

A distinctive aspect of the work by Pontejo et al. [127] is that the authors originally investigated vi ral TNFRs. The proposed modification of etanercept represents an application of fundamental knowl edge about viral proteins. In particular, it has been shown that the poxvirus protein CrmD can inhibit hTNF, mTNF, and murine LT, but not human LT. The study of the molecular basis of the differing inhibitory activity of the protein in relation to spe cies-specific ligands demonstrated that its inabil ity to inhibit human LT is due to the presence of a Glu–Phe–Glu motif in one of its structural loops. The importance of this motif in determining the receptor’s ability to bind to human LT was fur ther confirmed in studies of etanercept modifica tion [127].

**Fig. 9:**
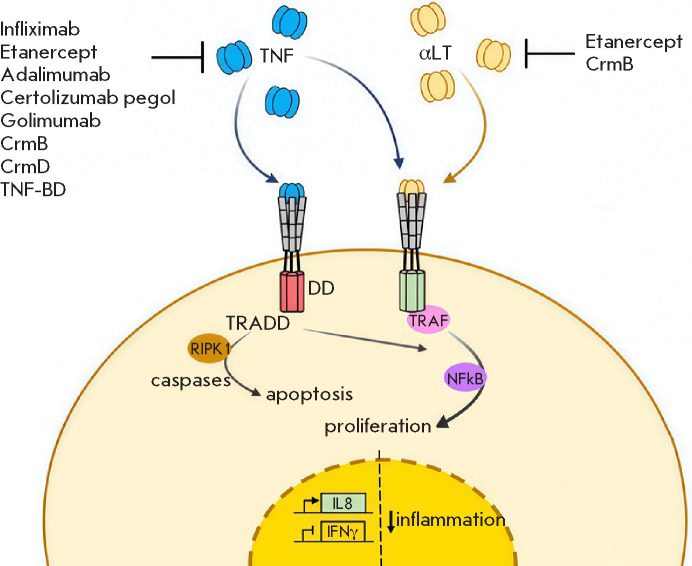
Schematic representation of the cellular response induced by the interaction of TNF and LT with their cellular re ceptors, and the molecular targets of clinically used TNF inhibitors [128], as well as orthopoxvirus TNF-binding proteins

Thus, anti-TNF agents engineered on the basis of orthopoxvirus proteins may become a promising al ternative to existing TNF inhibitors with a similar mechanism of action (Fig. 9).

## Abbreviations

TNF: tumor necrosis factor
RA: rheumatoid arthritis
TNFR: tumor necrosis factor re ceptor
ER: endoplasmic reticulum
PsA: psoriatic arthritis
DC: dendritic cell
p55: TNF receptor type I
p57: TNF receptor type II, CRD - cysteine-rich domain
PLAD: pre-ligand binding assembly domain
IL: interleukin
GM-CSF: granulocyte-macrophage colony-stimulating factor
Th1: T-helper cell type 1
LPS: lipopolysaccharide
CIA: collagen-induced arthritis
CPXV: cowpox virus
MPXV: monkeypox vi rus
VARV: variola virus
